# A Selective and Sensitive LC-MS/MS Method for Quantitation of Indole in Mouse Serum and Tissues

**DOI:** 10.3390/metabo12080716

**Published:** 2022-08-02

**Authors:** Vineet Joshi, Yashpal S. Chhonker, Dhruvkumar Soni, Kelly C. Cunningham, Derrick R. Samuelson, Daryl J. Murry

**Affiliations:** 1 Department of Pharmacy Practice and Science, College of Pharmacy, University of Nebraska Medical Center, Omaha, NE 68198, USA; vineet.joshi@unmc.edu (V.J.); dhruvsoniphd@gmail.com (D.S.); 2 Department of Internal Medicine–Pulmonary Division, College of Medicine University of Nebraska Medical Center, Omaha, NE 68198, USA; kelly.cunningham@unmc.edu (K.C.C.); derrick.samuelson@unmc.edu (D.R.S.); 3 Fred and Pamela Buffett Cancer Center, University of Nebraska Medical Center, Omaha, NE 68198, USA

**Keywords:** indole, LC-MS/MS, indole biodistribution, biomarker

## Abstract

Indole is an endogenous substance currently being evaluated as a biomarker for ulcerative colitis, irritable bowel syndrome, Crohn’s disease and non-alcoholic fatty liver disease. A novel, selective, and sensitive method using liquid chromatography coupled with tandem mass spectrometry (LC-MS/MS) was developed for quantitation of indole concentrations in mouse plasma and tissues. Samples were prepared by protein precipitation using ice-cold acetonitrile (ACN) followed by injecting the extracted analyte to LC-MS/MS system. Indole was separated using Synergi Fusion C18 (4 µm, 250 × 2.0 mm) column with mobile phase 0.1% aqueous formic acid (A) and methanol (B) using gradient flow with run time 12 min. The mass spectrometer was operated in atmospheric pressure chemical ionization (APCI) positive mode at unit resolution in multiple reaction monitoring (MRM) mode, using precursor ion > product ion combinations of 118.1 > 91.1 *m*/*z* for indole and 124.15 > 96.1 *m*/*z* for internal standard (IS) indole d7. The MS/MS response was linear over the range of indole concentrations (1–500 ng/mL). The validated method was applied for quantitation of indole concentrations range in mouse lungs (4.3–69.4 ng/g), serum (0.8–38.7 ng/mL) and cecum (1043.8–12,124.4 ng/g). This method would help investigate the role of indole as a biomarker and understand its implications in different disease states.

## 1. Introduction

Indole is an endogenous substance produced by numerous strains of microorganisms, including the genera *Escherichia*, *Pseudomonas* and *Aspergillus* [1], and is currently being evaluated as biomarker for a variety of disease states including ulcerative colitis, irritable bowel syndrome, Crohn’s disease and non-alcoholic fatty liver disease, among others [1]. Indole is biosynthesized in humans as a product of tryptophan metabolism in the intestine and, thus, helps maintain intestinal homeostasis. Intestinal bacteria alter pathways producing indole derivatives which in turn has concentration dependent effect on gut microbiota function [2].

A “biomarker” is a quantifiable, biological variable that characterizes cellular, tissue, physiological, pathological or clinical condition [3,4]. Eicosanoids, amino acids, bile acids, steroid hormones, nucleotides, vitamins, isoprenoids, etc., belong to the classes of endogenous compounds important to the maintenance of normal function [5]. Due to their significance to clinical research and drug discovery applications, accurate quantitative analysis of these endogenous analytes is essential [6,7,8]. Furthermore, comprehensive quantitation of endogenous biomarkers and their associated pathways assist in our understanding of their role in human health, disease pathophysiology and forensic investigations [9,10,11,12]. Additionally, biomarker quantitation aids in drug discovery process and, thereby, accelerates our efforts to improve quality of life in patient populations [13,14,15,16,17,18,19]. Indole plays an important role as biomarker in different organisms [20,21]. Additionally, the indolic structure forms a vital scaffold/pharmacophore assisting in various drug development processes with recent applications focused on developing lung cancer therapeutics [22]. Indole has been reported to be a fecal biomarker for the detection of clostridium difficile infections (CDI). Similarly, indole was identified as a susceptibility biomarker related to the development of cryptosporidium infections. Indole metabolites have been suggested to have clinical significance in certain disorders such as colorectal cancer, atherosclerosis and depression.

Metabolites derived from gut microbiota such as indole are reported to alter the intestinal barrier function and absorption of nutrients, thus interfering with the intestinal immune system. Indole and its metabolic derivatives are known to have a protective role in non-alcoholic fatty liver disease (NAFLD). In humans, indole concentration in the blood stream has been reported to be significantly lower among obese subjects compared to healthy subjects, along with increased fat content in liver for obese subjects. Indole stimulates production of mucin, which enhances the tight junction proteins, thus protecting the gut barrier function. Moreover, indole enhances expression of 6-phosphofructo-2-kinase/fructose-2,6 biphosphatase 3 (PFKFB3), which is responsible for the regulation of glycolysis, whereas it decreases the macrophage proinflammatory activation in PFKFB3-dependent manner [23]. In summary, lower concentrations of indole can lower glucose metabolism, which is directly related to obesity and higher fat content in liver. Thus, determining indole concentrations may help in assessing treatment effectiveness in these disorders [24]. Additionally, a panel of indole biomarkers quantitated by LC-MS/MS was reported to be a vital step forward in diagnosis and management of gastroentero-pancreatic neuroendocrine tumors [25]. Indole has also been associated with negative impairment of emotional behaviors in a rat model [26], and indole concentrations may be a biomarker for bacteremia treatment in humans [27]. Indole undergoes cytochrome-P450 mediated metabolism to indoxyl, a precursor to indoxyl sulfate which is a uremic toxin. High plasma and serum concentrations of indoxyl sulfate are associated with mortality in patients suffering from chronic kidney disease. Thus, a LC-MS method capable of determining blood and plasma concentrations of indole in patients with chronic kidney disease could help understand the risk of mortality in these patients [28,29,30].

To date, several analytical methods for indole and indole containing compounds are described in the literature including: spectrophotometry [31,32], high performance liquid chromatography (HPLC) [33,34], high pressure liquid chromatography mass spectrometry (LC-MS) [35], gas chromatography-tandem mass spectrometry (GC-MS/MS) [36], liquid chromatography mass spectrometry post solid phase extraction [37] and, most recently, ultraperformance liquid chromatography-tandem mass spectrometry (UPLC-MS/MS) [38,39,40,41,42]. We build upon these findings by developing and validating a selective and sensitive LC-MS/MS method for the quantitation of indole in mouse plasma and tissues. We demonstrate the application of the method for determining concentrations of indole in mouse serum and tissues.

## 2. Materials and Methods

### 2.1. Chemicals and Materials

Indole (purity: ≥99%) and indole d7 (IS) were purchased from Sigma-Aldrich (St Louis, MO, USA). LC-MS grade methanol, formic acid and acetonitrile were purchased from Thermo Fisher Scientific (Waltham, MA, USA). Ultrapure water was generated from a GenPUre xCAD plus water purification system (Thermo Fisher Scientific). Mouse serum and plasma was purchased from Equitech-Bio, Inc. (Kerrville, TX, USA). All other materials and reagents were purchased from standard chemical suppliers and were of analytical grade or higher.

### 2.2. Liquid Chromatographic and Mass Spectrometric Conditions (LC-MS/MS)

A Nexera LC-30AD LC-MS/MS system fitted with binary pump and autosampler (Nexera SIL-30AC) connected to Shimadzu LC-MS 8060 (Shimadzu Scientific Inc., Columbia MD, USA) with an APCI source and operated in positive mode was used for bioanalysis. Analyte separation was achieved using a Synergi Fusion C18 column (4 µm, 250 mm × 2.0 mm; Phenomenex, CA, USA) with Phenomenex C18 guard column. Mobile phase consisted of 0.1% formic acid in water (A) and methanol (B) with gradient flow program and total run time of 12 min was employed. The pump concentration was 50% B at 0 min, which was increased from 50 to 85% over 5 min, then constant at 85% B for 2 min and further increased from 85 to 95% B from 7–10 min and then reduced to initial conditions of 50% B over 0.1 min and held at initial conditions for 2 min prior to next injection. The column temperature was kept at room temperature and column pressure was 2000 psi, flow rate was maintained at 0.25 mL/min, and the injection volume was 10 µL. The MS was operated in positive APCI mode with MRM mode, using the precursor ion > product ion combination was found to be 118.15 > 91.1 *m*/*z* for indole and 124.15 > 96.1 *m*/*z* for indole d7.

For optimization of compound-dependent MS parameters, such as temperature, voltage, gas pressure, etc., the auto-optimization mode in the Shimadzu Labsolutions software (version 5.99) from Shimadzu Scientific Inc. was used. Indole (1 µg/mL) and indole-d7 IS (1 µg/mL) solution prepared in methanol was used for method development. Both indole and indole-d7 were more readily detected utilizing APCI positive ionization mode (data not shown). The following mass spectrometer source settings were utilized: nebulizer gas, 3.0 L/min; heating gas, 10 L/min; drying gas, 5 L/min; interface temperature, 300 °C; desolvation line temperature, 200 °C; and heat block temperature, 200 °C. The MRM transitions for each analyte and IS, as well as their respective optimum MS parameters, such as voltage potential (Q1, Q3), and collision energy (CE), are shown in Table 1. The Shimadzu Labsolutions LCMS software (Version 5.99) was used for acquisition and quantitation of MS data.

### 2.3. Preparation of Charcoal-Stripped Surrogate Matrix for Calibration Curves

Mouse plasma was treated with charcoal to adsorb and remove any endogenous indole and used for preparation of calibration standards. We have previously described that charcoal-treated plasma serves as a suitable blank matrix when endogenous compounds need to be quantitated [43]. The same principle was applied during development of this method. Briefly, charcoal suspension was prepared in Dulbecco’s phosphate buffer saline (PBS). An amount of 0.66 g charcoal (Fisher Chemical, Fairlawn, NJ, USA) was mixed with 100 mL of 0.1M PBS (pH = 7.4) using hot plate and stirrer. The resulting suspension was centrifuged at 4000× *g* for 15 min at 4 °C and the supernatant was discarded. A volume of 6 mL of mouse plasma was then added to the charcoal pellet and subjected to continuous mixing for 2 h. The mixture was then centrifuged at 13,000× *g* for 15 min at 4 °C and supernatant was collected. The process was repeated two times to ensure complete removal of endogenous substances.

### 2.4. Preparation of Stock, Calibration Standards and Quality Control Samples

Indole and indole-d7 (IS) were received as powder, which was dissolved in methanol separately to prepare 1 mg/mL stock solutions. These master stocks were further diluted using serial dilution to prepare indole 10 µg/mL and IS 1 µg/mL. The indole and indole-d7 stocks were used for preparation of working standards for calibration curve (CC). CCs were prepared by spiking 10 µL of working standard solution into 90 µL of charcoal-stripped surrogate matrix to achieve a concentration range of 1 to 500 ng/mL for indole. The concentrations for CC were 1, 2, 5, 10, 50, 200 and 500 ng/mL. Additionally, four Quality Control (QCs) were prepared including the Lower Limit of Quantification (LLOQ) sample, 1 ng/mL; Low QC, 3 ng/mL; Medium QC, 100 ng/mL; and High QC, 375 ng/mL. Samples were prepared in three replicates as per FDA’s guidance for Industry: Bioanalytical Method Validation (May 2018) [44] document. All CC and QCs stocks were stored at −20 °C until further use.

### 2.5. Plasma and Tissues Sample Preparation

Extraction of indole from plasma, serum, lungs and cecum was performed using protein precipitation method using ice cold ACN. All CCs and QC samples were spiked with 10 µL of indole working standard and 10 µL of indole-d7 internal standard (1 µg/mL). To this solution, 100 µL of serum or tissue homogenate was added, followed by 200 µL of acetonitrile (ice-cold, stored at −20 °C before use). The mixture was vortexed for 2 min and then centrifuged at 16,100× *g* at 4 °C for 10 min 150 µL of the supernatant solution was collected from the centrifuged solution and transferred to an auto-sampler vial and 10 µL injected into the LC-MS/MS system.

### 2.6. Assay Validation

The LC-MS/MS method was developed and validated in accordance with the current FDA guidelines (Guidance for Industry: Bioanalytical Method Validation-May 2018) [44].

Chromatograms of indole and IS-spiked sample were compared to study potential interference at the retention time for analyte and IS. This helped assess the specificity and selectivity of the method in blank mouse serum and charcoal-treated mouse plasma. Assay sensitivity was quantified using the signal-to-noise ratio (S/N) of each analyte in the CCs, and the limit of detection (LOD) and LLOQ were defined peak areas three and ten-fold greater S/N ratio, respectively. 

The concentrations used for generating calibration curve were 1, 2, 5, 10, 50, 100 and 500 ng/mL of indole in blank charcoal-stripped surrogate matrix along with indole-d7 as internal standard (1 µg/mL). Calibration curves were constructed by plotting the concentrations of indole on the x-axis and the peak area ratio (analyte/IS) of indole d7:IS on the y-axis. The lowest concentration (1 ng/mL) obtained from the calibration curve was set as the LLOQ. The analytical carry-over effects were evaluated by analyzing the blank matrix after three successive injections of high-level of QC samples. The acceptance criteria for each concentration in the calibration curve was set at ±15% of the expected value for standards and ±20% for LLOQ.

Inter-day and intra-day accuracy and precision was evaluated using four different QCs of indole in plasma. The QC concentrations used were 1 ng/mL (LLOQ), 3 ng/mL (LQC), 100 ng/mL (MQC) and 375 ng/mL (HQC). The QCs were analyzed along with standards in set of five replicates (n = 5) over a period of three days. The protein precipitation method described above was used for preparation of all standards and QCs. Similar to standards, the acceptance criteria for accuracy of each QC were set at ±15% of the expected concentration with the exception of LLOQ (±20% of expected concentration). Precision was calculated as the percent relative standard deviation (%RSD) of the expected concentration of the QC.

### 2.7. Recovery and Matrix Effect

For calculation of recovery, the concentration of plasma-extracted QCs was compared with blank extracts spiked with indole post-extraction. Extraction recovery was calculated for both indole and IS.

The matrix effect (*ME*) was calculated by comparing the peak area of indole concentrations of QCs post-extraction against those prepared in methanol (solvent used for reconstitution). Calculations for *ME* and *IS-normalized ME* was performed as per equations 1 and 2 mentioned below. If peak area ratio is less than 85% or greater than 115%, then matrix effect is assumed to exist. Otherwise, no matrix effect is considered [45].

(1) $$ME=\frac{Mean\;Peak\;area\;of\;analyte\;\mathrm{spiked}\;\mathrm{post}-\mathrm{extraction}\;}{Mean\;Peak\;area\;of\;analyte\;in\;Solvent}\;\times \;100$$

(2) $$IS\;normalized\;ME=\frac{Mean\;Peak\;area\;ratio\;of\;analyte/IS\;spiked\;post-extraction\;}{Mean\;Peak\;area\;ratio\;of\;analyte/IS\;in\;Solvent}\times 100$$

### 2.8. Stability

The stability of indole was assessed at LQC, MQC and HQC concentrations for three replicates. The stability in serum of analytes under −80 °C for 60 days (long-term stability) and 20 °C for 24 h (bench-top storage) were determined by comparing the mean peak areas ratio of analytes in respective conditions with the corresponding mean peak areas in freshly made. Auto sampler stability of analytes in processed was determined at 4 °C for 24 h.

## 3. Application of the Method and Sample Collection

Chronic alcohol consumption causes a myriad of negative health consequences, including alcohol-related liver disease (ALD), respiratory infections, neurological defects and pancreatitis. Many different mouse models of alcohol-feeding have been employed to evaluate injury, inflammation and fatty liver disease in patients suffering from chronic alcohol-related tissue injury. Binge-on-chronic alcohol feeding causes high blood-alcohol levels in mice and can be utilized to study alcohol liver disease and its impact on drug metabolism, as well as alcohol-related tissue injury [46]. However, the underlying mechanisms by which alcohol causes tissue injury are not completely understood, especially regarding changes in tissue and circulating levels of potentially bioactive metabolites (i.e., indole). Previously, we demonstrated that indole supplementation mitigated the risk for alcohol-associated bacterial pneumonia, and improved pulmonary NK cell numbers/recruitment in alcohol-fed mice [47].

Serum, cecal and lung samples were collected from all mice post their sacrifice by placing freshly isolated tissues directly into sterile 2 mL tubes. Serum, cecal content and lung samples were then flash frozen using liquid nitrogen and stored at −80 °C until further their analysis. Tissue samples were rinsed with phosphate buffered saline to diffuse blood out and were blotted with filter paper. Samples were then weighed and homogenized using a TissueLyser II (Qiagen Science, Louisville KY, USA) in de-ionized water. Cecal and lung samples were further diluted 5-fold with deionized water. Cecal and lung samples were stored at −80 °C until analysis. Serum (ng/mL) and tissue (ng/g) indole concentrations were determined for each time point collected.

## 4. Results and Discussion

The aim of this study was to develop and validate a selective and sensitive method for the quantitation of indole. For the purpose of this study, method development was subdivided into three parts: optimizing the MS parameters, LC parameters followed by selection of the best method for extraction of indole from biological matrix and subsequent quantitation.

### 4.1. Optimization of Mass Spectrometric Conditions

**Figure 1:**
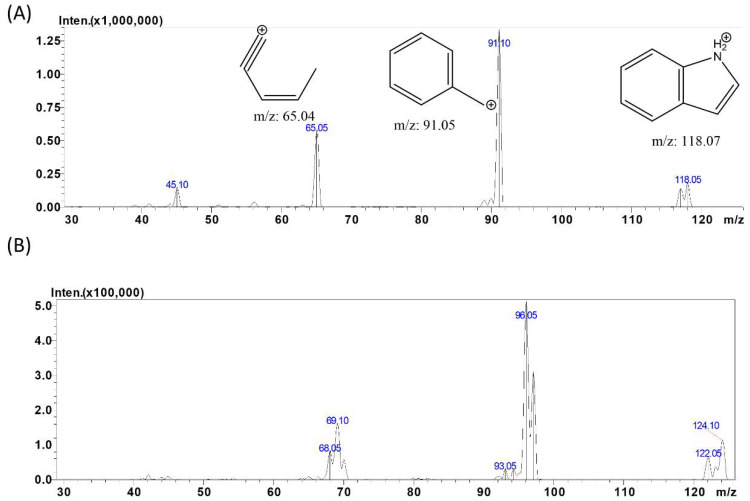
MS/MS product ion spectra of indole (**A**) in positive APCI mode [M + H], shows prominent fragments peak at *m*/*z* 91.1, 65.05, and 45.10 at CE-30 units and IS (**B**) shows prominent fragments peak at *m*/*z* 96.05 at CE-30 units.

To optimize mass spectrometric conditions, indole and indole-d7 at 1 µg/mL in methanol were prepared and injected into the LC-MS/MS system. APCI offers certain advantages, such as formation of single-charged ions, higher flow rates and ability to detect non-polar compounds and molecules that are not easily ionized. Since indole is a small molecule and non-polar in nature, APCI was employed for detection. Initial experiments comparing APCI and ESI as a source of ionization showed that ESI did not produce any signal, but produced a strong signal using APCI (data not shown). As a result, we decided to use APCI in our method. Using APCI in positive mode, the precursor ions (M + H) for indole and indole-d7 were obtained as 118.1 and 124.15 *m*/*z*, respectively. The fragmentation of indole and IS were selected using auto-optimization mode. For indole, the three most abundant fragments were found at 91.1, 65.05 and 45.10 *m*/*z* from highest to lowest intensity, respectively. Indole-d7 showed most abundant fragment at 96.05 *m*/*z*. The MRM fragmentation patterns for indole and indole-d7 are shown in Figure 1. The optimum collision energy using positive mode APCI was found to be −30 V. Based on these observations, it was concluded that APCI used along with MRM in positive mode provides high degree of selectivity for identification of indole.

### 4.2. Optimization of Chromatographic Conditions

**Figure 2:**
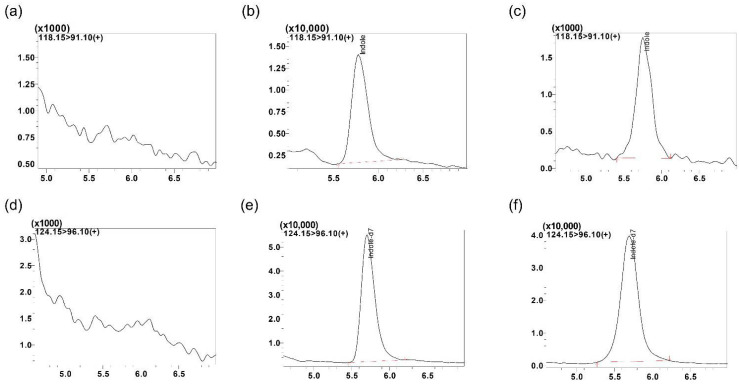
Representative MRM ion-chromatograms of (**a**) blank mouse lung homogenate using the conditions for indole detection, (**b**) indole spiked in mouse serum at 10 ng/mL (rt. 5.8 min), (**c**) indole mouse lungs homogenate study sample (rt. 5.8 min, Id: AI-2), (**d**) blank mouse lung homogenate using the conditions for indole-d7 detection, (**e**) indole-d7 spiked in mouse lung homogenate (rt. 5.8 min, 200 ng/mL) and (**f**) indole-d7 spiked in mouse study lung homogenate sample (Id: AI-2).

The goal for this section of method development was to achieve short retention time combined with improved peak shape and resolution for indole. Since indole is a small molecule and polar in nature, we used reversed phase chromatography and optimized the concentrations of both aqueous and organic mobile phase to achieve better separation of the compound. Briefly, 0.1% (*v*/*v*) acetic acid was used as the aqueous mobile phase (Mobile Phase A) along with methanol as organic mobile phase (Mobile Phase B) resulted in acceptable peak shape and retention time. The stationary phase column employed was Synergi Fusion C18 column (4 µm, 250 mm × 2.0 mm; Phenomenex, CA, USA) with Phenomenex C18 guard column. Retention time was found at 5.5 min was observed with good peak shape for indole, but no peak was found for the IS. Thus, we decided to replace 0.1% (*v*/*v*) acetic acid with 0.1% (*v*/*v*) formic acid. Flow rate was maintained at 0.25 mL/min and temperature at room temperature. A run time of 12 min and the mobile phase in gradient composition was employed (0–5 min—50% B (methanol) initially and then increased from 50 to 85% B (methanol) in 5–7 min. It was then further increased from 85 to 95% B (methanol) in 7–10 min, followed by a return to initial conditions (50% B), and maintained for 2 min to ensure equilibration of the column. Figure 2 shows chromatograms of indole in mouse lung homogenate. Figure 2a represents blank lung homogenate showing no peak for indole, whereas Figure 2b shows distinct peak for indole at 10 ng/mL in lung homogenate and Figure 2c represents indole peak in mouse study sample. Similarly, no peak is seen in Figure 2d for blank lung homogenate for detection of IS. Figure 2e shows distinct peak for IS spiked in lung homogenate at 200 ng/mL and Figure 2f shows mouse study sample lung homogenate spiked with IS at 200 ng/mL showing consistent retention time.

### 4.3. Selection of Extraction Method

Simple protein precipitation method using ice-cold acetonitrile was employed to isolate indole from mouse plasma, lungs, cecum and serum. This method was anticipated to provide easy and quick sample preparation compared to liquid–liquid extraction The mean extraction recoveries for indole and IS from mouse plasma, mouse serum and lungs for LQC, MQC and HQCs are shown in Table 2. Protein precipitation method provided consistent recovery across matrices evaluated. Since indole is an endogenous analyte, it is important to consider matrix effects for accurate quantitation. As a result, calibration curves should ideally be prepared in the same or an equivalent matrix to minimize matrix effects. One approach to eliminate matrix effect is to remove endogenous indole from the matrix by treating it with charcoal and removing the indole present in it. Using this approach, we prepared surrogate matrix by treating mouse plasma with charcoal for making the calibration curve. Using analyte/IS peak area ratios, the recoveries of indole in the charcoal-stripped matrix were similar to those in unstripped serum, and lungs (Table 2), indicating similar matrix effect for the study samples (unstripped matrix) and calibration curve (stripped plasma).

### 4.4. Assay Validation

#### 4.4.1. Specificity and Selectivity 

**Figure 3:**
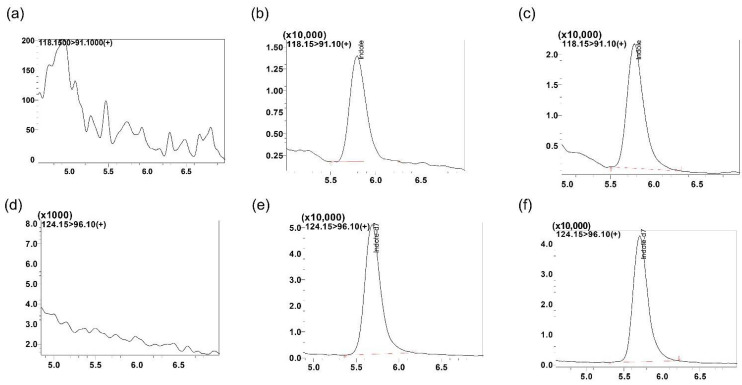
Representative MRM ion-chromatograms of (**a**) blank mouse serum using the conditions for indole detection, (**b**) indole spiked in mouse serum at 10 ng/mL (retention time, rt. 5.8 min), (**c**) indole mouse study serum sample (rt. 5.8 min, Id: AI-1_9-17-20), (**d**) blank mouse serum using the conditions for indole-d7 (IS) detection, (**e**) indole-d7 spiked in mouse serum (rt. 5.8 min, 200 ng/mL) and (**f**) indole-d7 spiked in mouse study serum sample (Id: AI-1_9-17-20).

The representative LC-MS/MS chromatogram with blank mouse serum and mouse study samples is shown in Figure 3. The retention times for indole and IS were 5.8 min.

#### 4.4.2. Calibration Curve and Linearity

Calibration curve was generated in mouse surrogate matrix. Linearity was observed in concentrations ranging from 1 to 500 ng/mL with r^2^ = 0.998. The lowest standard (1 ng/mL) showed RSD < 20% of expected concentrations. The ratio of signal to noise (S/N) was greater than ten and thus, the lowest standard was defined as LLOQ concentration (data not shown).

#### 4.4.3. Carry-Over

Blank samples were injected in sequence after the HQC and no significant peaks (≥ 20% of LLOQ) were observed, thus meeting the acceptance criteria (data not shown). Based on this, it was concluded that carryover would not be an issue with the method.

#### 4.4.4. Accuracy and Precision 

Four QCs (LLOQ, LQC, MQC and HQC) were used to assess accuracy and precision. Accuracy (% bias) ranged from 8.5 to −4.3% with intra-day variability of −4.3% at all concentrations. The precision (% RSD) ranged from 2.8 to 11.1% with LLOQ showing highest inter-day variability (Table 3). Both accuracy and precision were within the acceptable limits of ±15% of nominal concentrations and ±20% of the theoretical value at LLOQ. 

#### 4.4.5. Recovery and Matrix Effect 

The mean extraction recoveries of indole and IS at LQC, MQC and HCQ levels in plasma, serum and lung are provided in Table 2. Furthermore, the matrix effect and IS normalized matrix effect of not more than ±15% in different matrices were observed which are within the acceptance criteria. Our result confirmed that charcoal stripped plasma demonstrated similar recovery rates using analyte/IS peak area ratios.

#### 4.4.6. Stability

The different stability results are summarized in Table 4. Indole was found to be within ±15% of the actual concentration at the LQC, MQC and HQC concentrations. The results demonstrated indole’s stability under relevant storage conditions.

### 4.5. Application of the Method for Indole Quantitative Estimation

Chronic alcohol consumption can alter the gut and intestinal microbiota and their functions; however, very little knowledge about alcohol-associated dysbiosis on host cell against bacterial pneumonia is currently available. One of the approaches to reduce the bacterial burden and improve immune cell trafficking against bacterial pneumonia is treatment with tryptophan metabolites, such as indole or with probiotics. Thus, to study the protective effects of indole and investigate the role of microbiota-based therapy as a novel strategy for mitigating the risk of alcohol-associated bacterial pneumonia, it is important to measure endogenous indole concentrations [47]. The currently developed and validated method was used for quantitation of endogenous indole in mouse serum, cecum, and lungs tissue samples. Indole concentrations in mouse serum (0.8–38.7 ng/mL), lung (4.315–69.4 ng/g), and cecum (1043.8–12,124.4 ng/g) matrices were observed. We identified substantial variability in indole serum and tissue concentrations in mice using the developed method.

## 5. Conclusions

A selective, and sensitive LC-MS/MS method was developed and validated for the quantitation of indole in mouse bio-matrices. Using this method, indole was quantitated in mouse serum, cecum, and lungs samples. Linearity was demonstrated for concentrations over the range of 1 to 500 ng/mL (r^2^ = 0.998) and LLOQ was established as 1 ng/mL. The method provides a high degree of selectivity and sensitivity for quantitation of indole, which makes it ideal for high-throughput processing of analytical samples. Extraction of indole requires a small amount of tissue (100 µL of plasma or about 20 mg of tissue) which allows application of this method for quantifying indole from lower rodents like mice, which inherently have low body mass thereby putting constraints on samples volume. We demonstrated applicability of our method by quantitation of indole in mouse serum (0.8–38.7 ng/mL), lung (4.315–69.4 ng/g) and cecum (1043.8–12,124.4 ng/g) samples. Based on these findings, the described LC-MS/MS method provides a sensitive method to quantitate indole in a variety of biomatrices. The developed method can be utilized for quantitation of indole as a biomarker for identification for various diseases/disorders and could support our understanding of pathological and physiological role of indole in disease etiology and progression.

## Figures and Tables

**Table 1:** Summary of MS/MS parameters: precursor ion, fragment ions, voltage potential (Q1), collision energy (CE) and voltage potential (Q3) for indole and indole d7 IS.

Analytes	MRM Transition *m*/*z* (Q1→Q3)	Q1 (V)	Q3 (V)	CE (V)	Retention Time (min)
Target: Indole	118.15 > 91.1 ^a^	−15	−21	−24	5.8
	118.15 > 65.05	−15	−11	−34	
IS: Indole d7	124.15 > 96.1 ^a^	−10	−25	−24	5.8
	124.15 > 97.1	−11	−10	−25	

^a^ Transition used for quantitation; voltage: V; minute: min.

**Table 2:** Mean extraction recoveries of indole and indole-d7 in mouse charcoal striped plasma, mouse serum and mouse lungs.

Bio-Matrices	% Extraction Recoveries of Indole (Mean ± SD, n = 3)			% Extraction Recoveries of Indole-d7 (Mean ± SD, n = 6)
	LQC (3 ng/mL)	MQC (100 ng/mL)	HQC (375 ng/mL)	IS (200 ng/mL)
Mouse charcoal striped Plasma	107.2 ± 2.1	111.2 ± 1.8	102.0 ± 8.1	103.5 ± 1.6
Mouse Serum	106.7 ± 5.2	110.2 ± 8.2	102.5 ± 5.6	103.5 ± 1.6
Mouse Lungs	107.2 ± 14.5	102.3 ± 2.5	106.1 ± 12.5	103.6 ± 0.8

**Table 3:** Indole accuracy and precision. Intra-assay and inter-assay accuracy and precision of indole in mouse plasma (n = 5).

Nominal Conc. (ng/mL)	Accuracy		Precision	
	% Bias Intra–Assay	% Bias Inter–Assay	% RSD Intra–Assay	% RSD Inter–Assay
LLOQ (1 ng/mL)	5.4	−2.8	5.7	11.1
LQC (3 ng/mL)	2.5	−4.3	2.8	7.8
MQC (100 ng/mL)	7.9	8.5	9.7	7.8
HQC (375 ng/mL)	−4.3	3.5	7.4	9.2

**Table 4:** Stability of indole was tested in mouse plasma at different storage conditions, (Mean ± SD, n = 3).

Storage Conditions	Nominal Conc. (ng/mL)	% Accuracy
Bench-top (20 °C, up to 24 h)	LQC (3 ng/mL)	93.1 ± 7.8
	MQC (100 ng/mL)	105.4 ± 4.4
	HQC (375 ng/mL)	112.5 ± 1.8
Auto-sampler (AS) storage (4 °C, up to 24 h)	LQC (3 ng/mL)	103.2 ± 1.8
	MQC (100 ng/mL)	95.4 ± 5.3
	HQC (375 ng/mL)	102.5 ± 3.2
Long term stability (−80 °C, up to 60 days)	LQC (3 ng/mL)	103.2 ± 3.5
	MQC (100 ng/mL)	95.3 ± 2.5
	HQC (375 ng/mL)	92.1 ± 8.4

## Data Availability

Data is contained within the article.
